# Implementation of European Cross-border Electronic Prescription and Electronic Dispensing Service: Cross-sectional Survey

**DOI:** 10.2196/42453

**Published:** 2023-04-04

**Authors:** Reelika Jõgi, Johanna Timonen, Leena Saastamoinen, Ott Laius, Daisy Volmer

**Affiliations:** 1 Department of Marketing Authorisations The Estonian State Agency of Medicines Tartu Estonia; 2 School of Pharmacy University of Eastern Finland Kuopio Finland; 3 Information and Development Services The Finnish Medicines Agency Helsinki Finland; 4 The Estonian State Agency of Medicines Tartu Estonia; 5 Institute of Pharmacy Faculty of Medicine University of Tartu Tartu Estonia

**Keywords:** electronic prescription, ePrescription, cross-border ePrescription, electronic dispensing, eDispensing, eHealth, digital health, implementation, European Union

## Abstract

**Background:**

The European cross-border electronic prescription (CBeP) and cross-border electronic dispensing system was first implemented in January 2019 when it became possible to purchase medications from community pharmacies in Estonia using a Finnish ePrescription. In 2020, Estonian ePrescriptions became available to be dispensed in Finnish pharmacies. The CBeP is an important milestone in increasing access to medicines across the European Union, and it has been unstudied to date.

**Objective:**

This study aimed to investigate Estonian and Finnish pharmacists’ experiences of factors influencing access to, and dispensing of, CBePs.

**Methods:**

A web-based survey was conducted among Estonian and Finnish pharmacists between April and May 2021. The survey was distributed to all 664 community pharmacies (n=289, 43.5% in Estonia and n=375, 56.5% in Finland) where CBePs had been dispensed in 2020. The data were analyzed using frequencies and a chi-square test. Answers to open-ended questions were categorized using content analysis and then analyzed by frequency.

**Results:**

In total, 66.7% (84/126) of the responses from Estonia and 76.6% (154/201) of the responses from Finland were included in the study. The majority of Estonian (74/84, 88%) and Finnish (126/154, 81.8%) respondents agreed that CBePs have improved patients’ access to medications. Problems with the availability of medications when dispensing CBePs were reported by 76% (64/84) of the Estonian respondents and 35.1% (54/154) of the Finnish respondents. In Estonia, the most commonly reported availability problem concerned the same active ingredient (49/84, 58%) of the medication not being available in the market, whereas in Finland, the most common issue was the unavailability of equivalent package size in the market (30/154, 19.5%). Encountering ambiguities or errors in the CBePs was reported by 61% (51/84) of the Estonian respondents and 42.8% (66/154) of the Finnish respondents. Mostly, the availability issues and ambiguities or errors were encountered rarely. The most commonly encountered ambiguities or errors were incorrect pharmaceutical form (23/84, 27%) in Estonia and incorrect total amount of medication (21/154, 13.6%) in Finland. Technical problems with using the CBeP system were reported by 57% (48/84) of the Estonian respondents and 40.2% (62/154) of the Finnish respondents. Most of the Estonian and Finnish respondents (53/84, 63%, and 133/154, 86.4%, respectively) had access to guidelines for dispensing CBePs. More than half of the Estonian (52/84, 62%) and Finnish (95/154, 61.7%) respondents felt that they had received sufficient training on dispensing CBePs.

**Conclusions:**

Pharmacists in both Estonia and Finland agreed that CBePs improve access to medications. However, interfering factors, such as ambiguities or errors in CBePs and technical problems in the CBeP system, can reduce access to medications. The respondents had received sufficient training and were informed of the guidelines; however, they felt that the content of the guidelines could be improved.

## Introduction

### Background

Several new digital applications, such as telemedicine, electronic prescriptions (ePrescriptions), and electronic consultation, have been introduced in health care over the past 10 years. An ePrescription has commonly been defined as an accurate and understandable prescription that is issued by a prescriber electronically and transmitted with a minimal number of errors from the point of care to a pharmacy [1]. Electronic dispensing is the process of retrieving the prescription from an electronic database and dispensing the medication to the patient based on the retrieved ePrescription [2]. In both Estonia and Finland, electronic prescribing is used as a centralized and nationwide system for issuing and handling medical prescriptions. In Estonia, the system was introduced in 2010, and currently, a majority of prescriptions are issued electronically [3,4]. In Finland, electronic prescribing started with the first pilots in 2010, and it has been the sole prescription method since 2017 [5]. Both countries’ systems use a national electronic database (Prescription Center) for storing, processing, and issuing of ePrescriptions [1].

For more than a decade, citizens of the European Union have been able to use paper prescriptions to purchase medications from another member state. However, the overall number of dispensed cross-border prescriptions has been marginal. In a report published in 2012, it was calculated that cross-border paper prescriptions accounted for 0.02% to 0.04% (ie, approximately 2.3 million prescriptions per year) of all prescriptions dispensed in the European Union [6]. Furthermore, the mutual recognition of medications dispensed with cross-border paper prescriptions has been problematic. The report also stated that approximately 55% of the presented foreign paper prescriptions were not dispensed immediately. The main reason behind the deferment concerned problems with verification and authenticity.

The transition from paper prescriptions to ePrescriptions makes dispensing safer, more efficient, and more cost-effective [7,8]. A study carried out among Finnish pharmacists demonstrated that ePrescriptions have a positive effect on decreasing the number of prescription forgeries and dispensing errors and enhance the management of patients’ medications [9]. Similarly, a study conducted in Sweden showed that a majority of pharmacists found ePrescriptions safe and beneficial for patients and cost-effective for the pharmacy [10]. Conversely, if the ePrescription system is not of high quality and is poorly implemented, ePrescriptions can reduce the work efficiency of both pharmacists and physicians, endanger patient safety, and increase health care costs [7].

In 2011, Directive 2011/24/EU on patients’ rights in cross-border health care, which included a regulation on mutual recognition of prescriptions, was approved [11]. Under this directive, guidelines for an ePrescriptions data set for electronic exchange were developed in 2014 [12]. These guidelines include the technical and legal provisions of ePrescriptions, including data protection, patient safety, substitutions, and storage periods for health data. The eHealth Network project was launched in 2017 among 23 EU countries with the aim to ensure health care with increased quality and availability of medicinal products via electronic data. At the moment, 2 electronic cross-border health services are being introduced in the European Union under the brand MyHealth@EU: ePrescriptions and patient summaries [13].

The European cross-border ePrescription (CBeP) service was first implemented in January 2019 when it became possible for Finnish patients to purchase medications from community pharmacies in Estonia using Finnish ePrescriptions. In June 2020, Estonian ePrescriptions became valid in Finnish pharmacies. This made Estonia and Finland the first countries in the world where patients in both countries can buy medications with an ePrescription in either of the 2 countries. As of October 2022, the CBeP service is available in 6 EU countries: Estonia, Finland, Croatia, Portugal, Poland, and Spain; in addition, several countries are in the process of developing the cross-border exchange of ePrescriptions [13,14]. To obtain an ePrescription issued in country A (the country of prescription) from country B (the country of dispensing), the pharmacy in country B must register the patient’s ID document. The pharmacy in country B then retrieves the prescription data from country A with the patient’s consent and sends the dispensing information to country A via the national contact points for eHealth [15]. The patient needs to pay the full cost of the medication in the country of dispensation and can request reimbursement from their insurer in the country of residence [16,17].

The experiences of pharmacists and physicians who participated in piloting the European Patient Smart Open Services system concerning patient summaries and CBeP were investigated in 2014 [18]. However, to the best of our knowledge, there are no published studies on the practical experiences of the dispensing of CBePs yet. It is important to study the impact of this new health care application to determine whether it achieves the aim set by the European Union: to ensure access to safe and high-quality health care for all European citizens [11]. Pharmacists are the professionals who come into the closest contact with CBePs, which makes their experiences crucial in assessing the impact of the CBeP service.

### Objectives

The main aim of this study was to investigate the experiences of Estonian and Finnish pharmacists regarding the dispensing of CBePs, especially their experiences with the availability of medication and ambiguities or errors in CBePs, as well as experiences and problems with the use of the CBeP dispensing system and guidelines and training received to dispense CBePs.

## Methods

### Data Collection

A web-based survey was conducted among community pharmacists in Finland and Estonia from April 12, 2021, to May 3, 2021. An invitation to participate in the survey containing the link to the web-based questionnaire was sent to 46% (375/815) of the community pharmacies in Finland [19] and 58% (289/498) of the community pharmacies in Estonia [20] where at least 1 CBeP had been dispensed during 2020. The list of Estonian pharmacies was acquired from the Estonian Health Insurance Fund, and the invitation email was sent to the pharmacies by a researcher (RJ). The Social Insurance Institution of Finland sent the invitation email to Finnish pharmacies. The questionnaire was targeted at pharmacists who had experience in dispensing CBePs. The electronic survey was designed in, and distributed through, the LimeSurvey web-based survey tool (LimeSurvey GmbH) in Estonian and Finnish. Two reminders to fill in the web-based questionnaire were sent to the pharmacies via email.

### Questionnaire

The Estonian questionnaire included 37 questions, whereas the Finnish questionnaire included 38 questions (Multimedia Appendices 1 and 2). The Estonian questionnaire did not include the question about the sex of the respondent to maintain their anonymity.

The questionnaire was based on the official guidelines for dispensing CBePs [11,16] and some previous studies on ePrescriptions [6,7,17,18]. The questionnaire was created in English and translated into Estonian and Finnish by the researchers (Estonian: RJ; Finnish: LS and JT). Linguistic correspondence of the questionnaires was checked, and minor revisions were made as a result. In addition to the research team, 6 pharmacists from Finland and 7 pharmacists from Estonia piloted the questionnaire. Minor revisions were made as a result.

This paper reports the results of 6 parts of the questionnaire: received training and access to guidelines for dispensing CBePs (questions 2-4, 7, and 8), ambiguities or errors in CBePs (questions 15 and 16), pharmacists’ opinions on statements about CBeP (question 17), the availability of medications with CBePs (questions 19 and 20), experiences and technical problems with the CBeP system (questions 22-25), and background information (questions 1 and 32-36; Multimedia Appendices 1-3). From the question asking the pharmacists’ opinions on statements about the CBeP, only 2 statements are included in this paper: “The drug nomenclature is sufficient for CBePs” and “CBeP has improved patients’ access to medications.” This paper reports the results of 15 structured questions (questions 1-3, 7, 15, 16, 19, 20, 23, 24, and 32-36), 2 Likert-scale questions (questions 17 and 22), and 3 open-ended questions (questions 4, 8, and 25).

### Data Analysis

Only data from fully completed questionnaires were used for the data analysis. The statistical analyses were performed using SPSS software (version 28.0; IBM Corp). Both quantitative and qualitative analyses were used. Qualitative content analysis was used to categorize answers to open-ended questions. Descriptive statistics, including frequencies and cross tabulations, were used. Differences between and among Estonian and Finnish respondents were examined using the chi-square test. A difference of *P*<.05 was considered statistically significant. The answers to open-ended questions provided by the Finnish respondents were translated into English by a member of the research team (RJ), and a native Finnish-speaking member of the research team (LS) checked the accuracy of the translation.

The respondents who had not dispensed CBePs in 2020 (Estonia: 1/84, 1%; Finland: 27/154, 18%) were included in the analysis. The questionnaire was targeted at pharmacists who had experience in dispensing CBePs, and these pharmacists may have had experience in dispensing CBePs in 2019 (Estonian respondents) or in 2021 (Estonian and Finnish respondents). In the Likert-scale question about the sufficiency of drug nomenclature and access to medications, the response options *fully agree* and *somewhat agree* were combined, as were *fully disagree* and *somewhat disagree*.

### Ethics Approval, Participation, and Data Storage

The research ethics committee of the University of Tartu in Tartu, Estonia, approved the study (330/T-18). According to Finnish ethics instructions for research, this type of research does not require an ethics review [21]. Participating in this study was voluntary, and responding to the questionnaire and sending it to the researchers was regarded as informed consent to participate in the study. The data are stored in the cloud server of the University of Tartu with limited access (restricted by username and password).

## Results

### Study Population

In total, 327 questionnaires were returned (n=201, 61.5% from Finnish respondents and n=126, 38.5% from Estonian respondents). Of these 327 questionnaires, 47 (14.4%) from Finland and 42 (12.8%) from Estonia were partially filled in and therefore excluded from the study, leaving 238 (72.8%) for analysis. Of these 238 questionnaires, 154 (64.7%) were from Finland, and 84 (35.3%) were from Estonia.

The highest proportion of respondents were aged 30-39 years in Estonia (25/84, 30%) and 40-49 years in Finland (50/154, 32.5%; Table 1) and had worked in a community pharmacy for >20 years (30/84, 36% and 50/154, 32.5%, respectively). The Estonian respondents most commonly worked in Tallinn, the capital of Estonia (30/84, 36%); or south-eastern Estonia (22/84, 26%). Of the Finnish respondents, 18.8% (29/154) worked in Helsinki, the Finnish capital; 12.3% (19/154) worked in the surrounding metropolitan area (Espoo, Vantaa, or Kauniainen); and 29.9% (46/154) worked in other parts of southern Finland. A majority of Estonian and Finnish respondents had dispensed CBePs less than once a month in 2020 (51/84, 61% and 118/154, 76.6%, respectively). The Estonian respondents had dispensed CBePs more frequently than the Finnish respondents (*P*<.001).

**Table 1:** Study population characteristics of Estonian (N=84) and Finnish (N=154) respondents.

Characteristics		Estonian respondents, n (%)	Finnish respondents, n (%)
**Sex**			
	Female	N/A^a^	129 (83.8)
	Male	N/A	22 (14.3)
	Would not like to specify	N/A	3 (1.9)
**Age (years)**			
	≤29	16 (19)	29 (18.8)
	30 to 39	25 (29.8)	30 (19.5)
	40 to 49	15 (17.9)	50 (32.5)
	50 to 59	23 (27.4)	37 (24)
	≥60	5 (6)	8 (5.2)
**Position within the pharmacy^b^**			
	Dispenser or assistant pharmacist	17 (20.2)	91 (59.1)
	Pharmacist	31 (36.9)	45 (29.2)
	Pharmacy manager	28 (33.3)	4 (2.6)
	Pharmacy owner	8 (9.5)	14 (9.1)
**Length of employment in community pharmacy (years)**			
	<1	4 (4.8)	4 (2.6)
	1 to 5	13 (15.5)	28 (18.2)
	6 to 10	19 (22.6)	25 (16.2)
	11 to 20	18 (21.4)	47 (30.5)
	>20	30 (35.7)	50 (32.5)
**Frequency of dispensing CBePs^c^ by the pharmacists^d^**			
	No CBePs dispensed in 2020	1 (1.2)	27 (17.5)
	Less than once a month	51 (60.7)	118 (76.6)
	About once a month	14 (16.7)	7 (4.5)
	A few times a month	11 (13.1)	1 (0.6)
	About once a week	6 (7.1)	1 (0.6)
	Daily or almost daily	1 (1.2)	0 (0)

^a^N/A: not applicable (the Estonian questionnaire did not include the question about the sex of the respondent to maintain their anonymity).

^b^Staff in Finnish community pharmacies consist of the pharmacy owner or pharmacy manager; pharmacists; dispensers; and other pharmacy staff, such as pharmacy technicians. Staff in Estonian community pharmacies consist of the pharmacy owner and pharmacy manager; pharmacists; assistant pharmacists; and other pharmacy staff, such as customer assistants. A pharmacy owner, manager, and pharmacist have 5 years of education (MSc) at a university; a dispenser has 3 years of education (BSc) at a university; and an assistant pharmacist has 3 years of education (equal to a bachelor’s degree) at a higher education institution. Both pharmacists and dispensers or assistant pharmacists are licensed pharmacy practitioners who dispense medications independently and ensure the safe and proper use of medications among the public.

^c^CBeP: cross-border electronic prescription.

^d^Statistical significance was tested with a chi-square test (*P*<.001).

### Availability of Medication

Of the Estonian respondents, 73% (61/84) agreed that the drug nomenclature is sufficient for dispensing CBePs, whereas the corresponding proportion among the Finnish respondents was 77.3% (119/154). The share of respondents disagreeing with the statement was 24% (20/84) in Estonia and 12.3% (19/154) in Finland. The answer option *I don’t know* was selected by 4% (3/84) of the Estonian respondents and 10.4% (16/154) of the Finnish respondents. Of the Estonian and Finnish respondents, 88% (74/84) and 81.8% (126/154), respectively, agreed that CBePs have improved patients’ access to medications, whereas 5% (4/84) of the Estonian respondents and 7.8% (12/154) of the Finnish respondents disagreed with the statement. The answer option *I don’t know* was selected by 7% (6/84) of the Estonian respondents and 10.4% (16/154) of the Finnish respondents.

The problems with CBePs in terms of the availability of medication were more common in Estonia than in Finland (*P*<.001; Table 2): 76% (64/84) of the Estonian respondents and 35.1% (54/154) of the Finnish respondents had encountered problems with availability of medication. However, most of the respondents who had encountered problems had encountered them rarely (89/118, 75.4%). The availability problems regarding the same active ingredient (*P*<.001) and strength of the corresponding medication (*P*<.001) were more common in Estonia than in Finland, and more than half of the Estonian respondents had witnessed such problems (49/84, 58% and 48/84, 57%, respectively; Table 3). The most reported availability problem among Finnish respondents was the size of the corresponding medication’s package not being available in the market (30/154, 19.5%).

**Table 2:** Estonian (N=84) and Finnish (N=154) respondents’ experiences with interfering factors in dispensing cross-border electronic prescriptions (CBePs).

Interfering factors				Always, n (%)		Often, n (%)		Rarely, n (%)		Never, n (%)		*P* value	
**Problems with** **CBePs** **in terms of the availability of medication**													<.001
	Estonian respondents		0 (0)		19 (22.6)		45 (53.6)		20 (23.8)				
	Finnish respondents		0 (0)		10 (6.5)		44 (28.6)		100 (64.9)				
**Ambiguities or errors in** **CBePs**													.003
		Estonian respondents	0 (0)		15 (17.9)		36 (42.9)		33 (39.3)				
		Finnish respondents	8 (5.2)		17 (11)		41 (26.6)		88 (57.1)				
**Technical problems in the** **CBeP system**													<.001
		Estonian respondents	0 (0)		21 (25)		27 (32.1)		36 (42.9)				
		Finnish respondents	7 (4.5)		35 (22.7)		20 (13)		92 (59.7)				

**Table 3:** Problems with the availability of medication and ambiguities or errors in cross-border electronic prescriptions (CBePs) reported by Estonian (N=84) and Finnish (N=154) respondents.

		Estonian respondents, n (%)^a^		Finnish respondents, n (%)^a^		*P* value	
**Problems with** **CBePs** **in terms of the availability of medication**							
	Medication with the same active ingredient unavailable in the market		49 (58.3)		17 (11)		<.001
	Medication with the same strength unavailable in the market		48 (57.1)		16 (10.4)		<.001
	Equivalent package size unavailable in the market		29 (34.5)		30 (19.5)		.48
	Medication with the same formulation unavailable in the market		27 (32.1)		12 (7.8)		.02
	Pharmacy currently does not have the medication in stock		22 (26.2)		13 (8.4)		.22
	Other^b^		6 (7.1)		1 (0.6)		N/A^c^
**Ambiguities or errors in CBePs**							
	Incorrect pharmaceutical form		23 (27.4)		8 (5.2)		<.001
	Incorrect strength		21 (25)		13 (8.4)		.01
	Differences in ATC^d^ codes between countries^e^		17 (20.2)		17 (11)		.37
	Incorrect total amount of medication		15 (17.9)		21 (13.6)		.78
	Unclear or incorrect dosage instructions		14 (16.7)		19 (12.3)		.87
	Missing dosage instructions		12 (14.3)		4 (2.6)		.006
	Incorrect medication		3 (3.6)		5 (3.2)		.72
	Weight of child (aged <12 years) missing		2 (2.4)		1 (0.6)		.42
	Missing notation of exceptional dosage instructions or exceptional purpose of use		N/A		3 (1.9)		N/A
	Other^f^		10 (11.9)		18 (11.7)		N/A

^a^Respondents may have chosen several answer options.

^b^Other examples were related to limitations in dispensing cross-border electronic prescriptions and patients’ knowledge on cross-border electronic prescriptions.

^c^N/A: not applicable.

^d^ATC: anatomical therapeutic chemical.

^e^Differences in anatomical therapeutic chemical codes occur because Estonia uses unique anatomical therapeutic chemical codes for combination products (information received from O Laius, PhD [email, February 10, 2021]).

^f^Other examples were mostly related to availability of medications and limitations in dispensing cross-border electronic prescription*s* (eg, psychotropic drugs).

### Ambiguities or Errors in CBePs

In all, 61% (51/84) of the Estonian respondents and 42.8% (66/154) of the Finnish respondents had encountered ambiguities or errors in the CBePs (Table 2). However, most of them had encountered ambiguities or errors rarely (77/117, 65.8%). These problems were more common in Estonia than in Finland (*P*=.003). The most commonly encountered ambiguities or errors among Estonian respondents were related to the pharmaceutical form (23/84, 27%) and strength of the medication (21/84, 25%), whereas the most common ambiguities or errors reported by Finnish respondents were issues related to the total amount of medication (21/154, 13.6%; Table 3). Compared with the Finnish respondents, the Estonian respondents encountered more problems with incorrect pharmaceutical form (*P*<.001), incorrect strength (*P*=.01), and missing dosage instructions (*P*=.006).

### Technical Problems in the CBeP System

In all, 57% (48/84) of the Estonian respondents and 40.2% (62/154) of the Finnish respondents had encountered technical problems in the CBeP dispensing system that hindered or slowed the dispensing of a CBeP (Table 2). Technical problems were reported more frequently by the Estonian respondents than the Finnish respondents (*P*<.001). Of the Estonian and Finnish respondents who had encountered technical problems in the CBeP system, 88% (42/48) and 82% (51/62), respectively, had been unable to dispense a CBeP because a problem occurred. Respondents who had encountered technical problems (Estonian respondents: 48/84, 57%, and Finnish respondents: 62/154, 40.2%) detailed them in an answer to an open-ended question. The most commonly reported problems among Estonian (22/48, 46%) and Finnish (37/62, 60%) respondents were caused by maintenance work or connection problems. Some of the Finnish respondents mentioned problems with correcting (4/62, 6%) or canceling (3/62, 5%) the dispensing of an opened prescription, and some of the Estonian respondents (12/48, 25%) reported technical problems, which, in fact, were related to regulative issues, such as the *not dispensable* message in the prescription.

The pharmacists were also asked to describe the solutions to the problems that occurred. Of those pharmacists who experienced technical problems, 42% (20/48) of the Estonian respondents and 45% (28/62) of the Finnish respondents confirmed that the problems that had arisen had been resolved, either completely or partially. Additionally, 40% (19/48) of the Estonian respondents and 29% (18/62) of the Finnish respondents did not know whether the problems described had been resolved. The rest of the pharmacists—19% (9/48) of the Estonian respondents and 26% (16/62) of the Finnish respondents—reported that the problems had not been resolved or were recurring.

The majority of the respondents in Estonia (80/84, 95%) and Finland (137/154, 89%) knew where to get technical help from. Most of them—65% (52/80) of the Estonian respondents and 82.5% (113/137) of the Finnish respondents—received help from the IT helpdesk of the pharmacy dispensing system operator. Furthermore, 23% (18/80) of the Estonian respondents and 10.2% (14/137) of the Finnish respondents mentioned the national contact points for eHealth as their source of technical help.

### CBeP Dispensing Systems in Use

**Figure 1:**
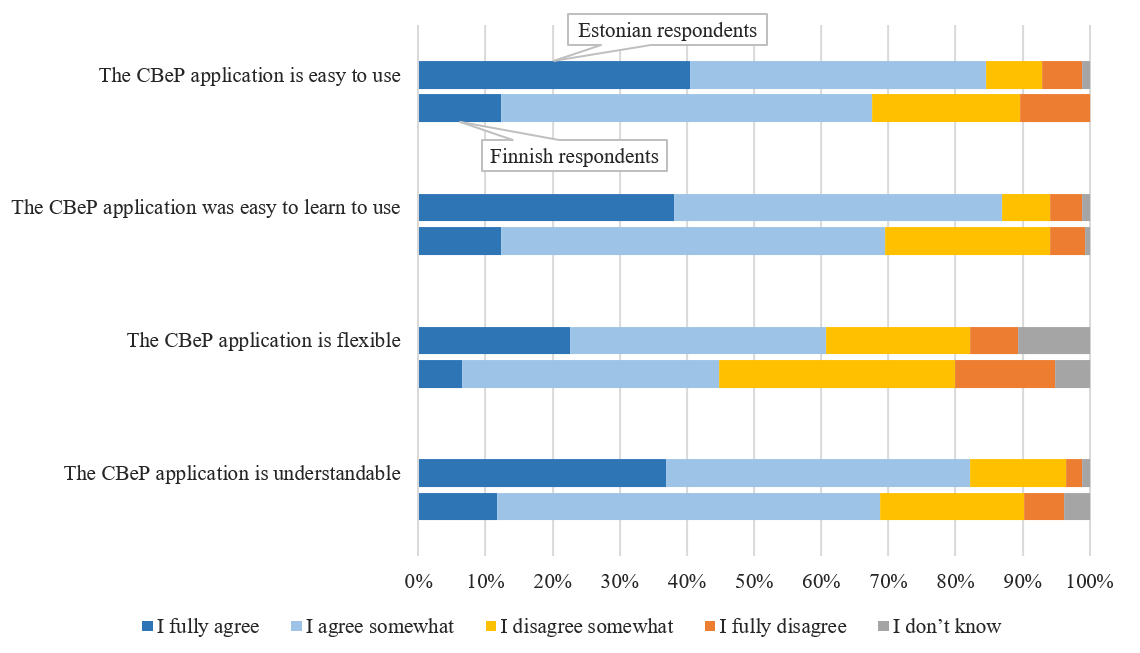
Experiences of Estonian (N=84) and Finnish (N=154) respondents in using the cross-border electronic prescription (CBeP) dispensing application.

Most of the Estonian and Finnish respondents felt that the CBeP dispensing application is easy to use (175/238, 73.5%), easy to learn to use (180/238, 75.6%), and understandable (175/238, 73.5%; Figure 1). Compared with the Finnish respondents, the Estonian respondents more often reported the CBeP application to be easy to use (*P*<.001), easy to learn to use (*P*<.001), flexible (*P*<.001), and understandable (*P*<.001). The Finnish respondents were more dissatisfied with the flexibility of the CBeP application than the Estonian respondents. No statistically significant differences were found between the applications used within the 2 countries.

### CBeP Dispensing Training

Most of the respondents in Estonia (52/84, 62%) and in Finland (95/154, 61.7%) reported receiving sufficient training on dispensing CBePs. Moreover, 19% (16/84) of the Estonian respondents and 27.9% (43/154) of the Finnish respondents reported that their training had been insufficient, and 19% (16/84) of the Estonian respondents and 10.4% (16/154) of the Finnish respondents reported not receiving any training. The Estonian respondents had received training more often via email or paper than the Finnish respondents (*P*<.001; Table 4). Compared with the Estonian respondents, the Finnish respondents had received training more often by independent information retrieval (*P*<.001), training videos (*P*<.001), and web-based seminar (*P*<.001).

**Table 4:** Types of training on dispensing cross-border electronic prescriptions received by Estonian (N=84) and Finnish (N=154) respondents.

Type of training	Estonian respondents, n (%)^a^	Finnish respondents, n (%)^a^	*P* value
Instructions via email or paper	59 (70.2)	32 (20.8)	<.001
Independent retrieval of information	13 (15.5)	64 (41.6)	<.001
Face-to-face seminar	13 (15.5)	N/A^b^	N/A
Training videos	7 (8.3)	50 (32.5)	<.001
Web-based seminar	5 (6)	39 (25.3)^c^	<.001
PowerPoint slides from THL^d^ and Kanta services^e^	N/A	58 (37.7)	N/A
Other^f^	4 (4.8)	19 (12.3)	N/A

^a^Respondents may have chosen several answer options.

^b^N/A: not applicable.

^c^Web-based seminar by the Finnish Institute for Health and Welfare.

^d^THL: Terveyden ja hyvinvoinnin laitos (Finnish Institute for Health and Welfare).

^e^Kanta produces digital health care and social welfare services for health care professionals, pharmacies, and citizens in Finland.

^f^Other examples included unspecified instructions from pharmacy or pharmacy information system operator and guidance from a colleague.

No statistically significant differences were found in either country in the type of training received among respondents who reported receiving sufficient or insufficient training, with 1 exception: compared with those who did not receive sufficient training (4/43, 9%; *P*<.001), the Finnish respondents who received sufficient training were more likely to have received training via a web-based seminar provided by the Finnish Institute for Health and Welfare (35/95, 37%). There were no statistically significant differences between the study population characteristics and sufficiency of the training received.

Responding to the open-ended question on training needs, the Estonian respondents (16/84, 20%) expressed a need for more training on topics related to regulations and dispensing. The respondents wanted to know more about the differences in regulations between the 2 countries and, more specifically, the differences in the amount of medication that can be dispensed at once. Similarly, the Finnish respondents (43/154, 27.9%) expressed a need to learn more about topics related to regulations and dispensing. In their answers, the respondents said they needed more knowledge on the validity of prescriptions and the type of medications, including quantities, that can be dispensed with CBePs. In addition, the Finnish respondents mentioned patient-related topics, such as identifying social security numbers or identity numbers of patients from different countries and informing patients about the regulations and compensation with regard to CBePs. The Finnish respondents also expressed a need for practical guidelines for dispensing CBePs in the CBeP dispensing system. The respondents added that although there are different guidelines available, they are not suitable for in-process use because they are too lengthy or have been provided in an inconvenient format.

### Guidelines for Dispensing CBePs

Of the Estonian respondents, 63% (53/84) had access to guidelines for dispensing CBePs, 31% (26/84) did not know whether they had access, and 6% (5/84) did not have access to the guidelines. Of the Finnish respondents, 86.4% (133/154) had access to the guidelines, 11% (17/154) did not know whether they had access, and 2.6% (4/154) did not have access to the guidelines. The Finnish respondents had access to the guidelines more often than the Estonian respondents (*P*<.001).

The open-ended question about guidelines available for the pharmacists was answered by 63% (53/84) of the Estonian respondents and 86.4% (133/154) of the Finnish respondents. The Finnish respondents most frequently mentioned the guidelines prepared by the pharmacy information system operator (Maxx, Salix, and PD3; 85/154, 55.2%) and the pharmacy’s own instructions (37/154, 24%; Table 5). The Estonian respondents most often mentioned guidelines of unknown origin (29/84, 34.5%), such as printed guidelines or instructions via email.

**Table 5:** Guidelines for dispensing of cross-border electronic prescriptions mentioned by Estonian (N=84) and Finnish (N=154) respondents.

Guidelines available	Estonian respondents, n (%)^a^	Finnish respondents, n (%)^a^
Pharmacy information system operator’s guidelines	14 (16.7)	85 (55.2)
Pharmacy’s own instructions	7 (8.3)	37 (24)
Guidelines by Kanta services^b^ and THL^c^	1 (1.2)	30 (19.5)
Not specified	29 (34.5)	15 (9.7)
Guidelines by TEHIK^d^	8 (9.5)	N/A^e^
Other^f^	1 (1.2)	13 (8.4)

^a^Respondents may have reported several guidelines.

^b^Kanta produces digital health care and social welfare services for health care professionals, pharmacies, and citizens in Finland.

^c^THL: Terveyden ja hyvinvoinnin laitos (Finnish Institute for Health and Welfare).

^d^TEHIK: Tervise ja Heaolu Infosüsteemide Keskus (Estonian Health and Welfare Information Systems Center).

^e^N/A: not applicable.

^f^Finnish respondents mentioned guidelines by the Association of Finnish Pharmacies, the Social Insurance Institution of Finland, the Finnish Medicines Agency, and the Patient Information Notices. Estonian respondents mentioned guidelines by the Estonian State Agency of Medicines.

## Discussion

### Principal Findings

To our knowledge, this is the first study exploring pharmacists’ practical experiences with CBePs. In the responding pharmacists’ opinion, CBePs improve access to medications. However, problems with the availability of medication and ambiguities or errors in CBePs emerged occasionally. In addition, technical problems with the CBeP dispensing system sometimes prevented the dispensing of medication. The pharmacists found the CBeP dispensing system to be easy to use, easy to learn to use, and understandable, but they encountered problems related to the inflexibility of the system. Most of the respondents (147/238, 61.8%) felt that they had received sufficient training on dispensing CBePs. The study also showed that the pharmacists were well informed on the available guidelines for dispensing CBePs. However, the pharmacists expressed the need for more information on regulations regarding the prescribing and dispensing of CBePs and more compact guidelines for dispensing CBePs.

CBePs improve access to medications because patients are able to purchase prescription medications abroad with ePrescriptions issued in their home country at any time, with some limitations. Since the beginning of 2019, >20,000 CBePs have been dispensed, the majority of which were dispensed in either Estonia or Finland [22]. The results of this study support improved accessibility to medications. However, this study brought out several factors that potentially interfere with the dispensing of CBeP medications and, therefore, may affect access to medications. One of these factors is the unavailability of medications. In addition to the differences in the selection of active ingredients in the market, different strengths, package sizes, and dosage forms can also become obstacles for the patient with regard to receiving the needed medication. Medication availability problems were reported more commonly in Estonia than in Finland. This study did not reveal the reasons behind this finding; therefore, it should be studied further. Even so, most of the respondents (180/238, 75.6%) in both countries agreed that the drug nomenclature is sufficient for dispensing CBePs. It should be noted that generic substitution of medications is allowed in both countries. This means that prescribed medication can be substituted with an interchangeable medication containing the same active ingredient. A study carried out in Finnish community pharmacies showed that medicine shortages seldom caused problems in pharmacies because generic substitution enabled pharmacists to substitute the patient’s medicine with an available interchangeable product [23]. In general, permission for generic substitution is available or even mandatory in most European countries, with some exceptions, such as Austria, where generic substitution is prohibited [24]. As there are differences in the available medications among countries, generic substitution should be encouraged in the European Union to improve cross-border access to medications. At the moment, there is no common set of rules across the European Union regarding the substitution of medications [25].

Access to medications is also affected by ambiguities or errors in ePrescriptions. In addition to potentially accidental errors, such as incorrect pharmaceutical form or strength, the respondents of this study had encountered problems related to regulative differences, such as differences in the anatomical therapeutic chemical codes among countries. Estonia uses unique anatomical therapeutic chemical codes for combination products (information received from O Laius, PhD [email, February 10, 2021]), which might explain the differences. The problems with incorrect total amount of medication could be caused by the differences in prescribing practices. However, Finnish pharmacists dealing with Finnish ePrescriptions also commonly encountered such ambiguities or errors [9]. The respondents also reported having problems with unclear or incorrect dosage instructions. A previous study [26] investigating the anomalies in ePrescriptions in Finland found anomalies related to dosage instructions to be the most frequently reported issues. Therefore, it is essential that the quality of prescribing practices is improved domestically. Furthermore, the dosage instructions in CBePs are written in the patient’s native language, and they are not translated in the system. This increases the workload of pharmacists if, in addition, the dosing instructions are not clearly written by the physician (eg, use of abbreviations and typographical errors). This might also compromise medication safety.

This study showed that access to medications was moderately affected by technical issues. Of the 110 respondents who had faced technical problems, 93 (85%) had been unable to dispense a CBeP because of such problems at least once. This shows that access to medications can be significantly reduced in the event of a technical problem. However, the Estonian respondents categorized the *not dispensable* message in the prescription as a technical issue, when, in fact, it is related to CBeP dispensing regulations; for example, it is not possible to dispense narcotics, psychotropic medications, extemporaneous medications, medications unauthorized in the country of origin, combination packages containing several different preparations, patient-specific special licensed medicines, and medical devices with CBePs [16,17]. Furthermore, Finnish ePrescriptions prescribed for a specific period (eg, *one year’s supply*) cannot be dispensed abroad. Such prescriptions are listed with a *not dispensable* message when a pharmacist retrieves the customer’s ePrescription information. Pharmacists’ lack of knowledge on the *not dispensable* message indicates that they may not have received the necessary guidelines for CBeP regulations and that the in-system information provided for pharmacists is too limited. The more pharmacists have to deal with technicalities and unknown issues, the less time they have for providing quality patient counseling. It has been shown that poorly implemented ePrescription systems lower work efficiency, threaten patient safety, and increase health care costs [7].

In addition to technical problems, this study explored user satisfaction with the CBeP dispensing systems. Both countries’ systems work similarly in terms of information retrieval but use different software systems. In Estonia, 2 main pharmacy software systems are being used: NOOM and Hansasoft. The main pharmacy software systems used in Finland are Maxx, PD3, and Salix. The CBeP dispensing applications therefore differ not only between but also within the 2 countries. In general, the respondents found the systems easy to use, easy to learn to use, and understandable but inflexible. The mainly positive user experiences are consistent with the experiences of pharmacists who evaluated the European Patient Smart Open Services system (the piloting project for CBePs and patient summaries) [18]. The dissatisfaction with the level of flexibility was more prevalent among the Finnish respondents, which could be due to the fact that Estonian pharmacists have been dispensing CBePs longer and are familiar with the system. Another factor—perhaps the most prevalent—for causing dissatisfaction with the flexibility could be the difficulty encountered by Finnish pharmacists in making corrections to, or canceling dispensing, the CBeP after opening the ePrescription. If a pharmacist has to close an ePrescription temporarily in the middle of dispensing because of problems with the connection or other technical issues, the ePrescription will be lost and cannot be dispensed again. Similar problems were described by a study conducted in Finland, where the inflexibility of the ePrescription dispensing system was caused by the difficulty in correcting and modifying ePrescriptions during dispensing [27].

Although most of the respondents (147/238, 61.8%) had received sufficient training on dispensing CBePs, 38.2% (91/238) of the respondents felt that they had received insufficient training or no training at all. In both countries, the official training was planned to be provided on the web without face-to-face meetings. It is essential that the training for dispensing CBePs be accessible at all times, both for new pharmacists and for refreshing pharmacist knowledge as most of the pharmacists (169/238, 71%) had dispensed CBePs less than once a month. Hence, guidelines need to be available to pharmacists, and all pharmacists have to be aware of such guidelines. The Finnish respondents, although better informed about the guidelines than the Estonian respondents, expressed a need for more practical and compact technical guidelines for dispensing CBePs. For the pharmacists to be able to identify problems and, when necessary, advise patients and physicians on how to proceed with nondispensable ePrescriptions, it is essential that the guidelines include information on the regulations and national specificities regarding the issuing of ePrescriptions. These guidelines should also be available to physicians to avoid problems when prescribing medications.

### Strengths and Limitations

The data were collected with a uniform questionnaire from pharmacists in 2 countries simultaneously. The survey was targeted at, and sent to, all pharmacies in Estonia and Finland that had experience in dispensing CBePs. As a result, we ensured that the right target group had been reached.

The main limitation of this study is that it was not possible to determine the response rate or estimate the representativeness of the responding pharmacists with regard to the target population. To maintain the anonymity of the respondents, they were not asked to disclose the name of the pharmacy they worked at. Furthermore, there is no information available on the number of pharmacists in different pharmacies or how many of the pharmacists have dispensed CBePs. Most of the respondents (169/238, 71%) had dispensed CBePs less than once a month; therefore, they may have limited experience in dispensing CBePs. This might be explained by the study period (2020) and the launch of the questionnaire (April-May 2021) coinciding with the COVID-19 pandemic, which limited traveling and, therefore, the number of dispensed CBePs. The timing may also have affected the answers to, and willingness to answer, the questionnaire. Estonian pharmacists have been dispensing CBePs longer than Finnish pharmacists (since January 2019 and since June 2020, respectively), which might affect the emphasis of the problems raised. In further studies, the questionnaire could be improved by including a time period (eg, *the last 6 months*) for the respondents to base their answers on.

### Conclusions

Estonian and Finnish pharmacists agree that CBePs improve access to medications. However, interfering factors, such as the unavailability of medication, ambiguities or errors in the CBePs, and technical problems in the CBeP dispensing system, may adversely affect access to medications. To maintain the quality of dispensing CBePs and to help pharmacists and physicians provide information about CBePs to patients, accessible high-quality guidelines and training on dispensing CBePs must be made available.

## Appendix

Multimedia Appendix 1 The Estonian version of the study questionnaire for pharmacists regarding cross-border electronic prescriptions.

Multimedia Appendix 2 The Finnish version of the study questionnaire for pharmacists regarding cross-border electronic prescriptions.

Multimedia Appendix 3 The English version of the study questionnaire for pharmacists regarding cross-border electronic prescriptions.

## Abbreviations

CBeP: cross-border electronic prescription
ePrescription: electronic prescription
