# Supplementary material for: Implementation of European Cross-border Electronic Prescription and Electronic Dispensing Service: Cross-sectional Survey
Source: J Med Internet Res. 2023 Apr 4;25:e42453. doi: 10.2196/42453 (PMC10132001; doi:10.2196/42453)
Supplement: Multimedia Appendix 1 [file jmir_v25i1e42453_app1.docx]

**Appendix 1 - Piiriülese digiretsepti küsimustik proviisoritele ja farmatseutidele**

Lugupeetud proviisorid ja farmatseudid

Tere tulemast uuringusse „Piiriülene digiretsept – esimesed tulemused Eestist ja Soomest“ (Cross-border e-prescribing and e-dispensing – the first results from Estonia and Finland). Antud küsimustik on suunatud **ainult** **neile proviisoritele ja farmatseutidele**, **kellel on kogemusi piiriülese digiretsepti alusel ravimite väljastamisega.**

Vastavalt direktiivile 2011/24/EL, mis käsitleb patsientide õigusi piiriüleses tervishoius, saavad Euroopa Liidu kodanikud kasutada ohutut ja kvaliteetset tervishoiuteenust kõikides Euroopa Liidu riikides ning saada selle eest ka kodumaal hüvitatud. 2014. aastal loodi selle direktiivi põhjal suunised digiretseptide andmevahetuseks liikmesriikide vahel. Retseptiandmete vahetus on osa projektist, mille rakendamisega alustasid 2017. aastal 23 Euroopa Liidu liikmesriiki. Projekti eesmärgiks on tagada ravimite parem kättesaadavus ja tervishoiukvaliteet elektroonse andmevahetuse kaudu.

**Antud küsimustiku eesmärgiks on uurida proviisorite ja farmatseutide kogemusi ravimite väljastamisel piiriülese digiretsepti alusel ning analüüsida selle mõju ravimite kättesaadavusele ja ohutule kasutamisele.** Uuringu tulemusi saab kasutada töötamaks välja ühtset piiriüleste digiretseptide süsteemi. Projektis osalevad nii Eesti kui ka Soome proviisorid ja farmatseudid. Küsitluse läbiviimist rahastab Kansaneläkelaitos (Kela) ehk Soome Sotsiaalkindlustusamet ning uuring viiakse läbi Tartu Ülikooli, Ida-Soome Ülikooli, Kela ja Eesti Ravimiameti koostööna.

Küsimustiku täitmine võtab aega umbes 15 minutit. Vastake küsimustele, valides kõige sobivama(d) vastuse(d) või kirjutage vastus selleks ettenähtud lahtrisse.

Vastused kogutakse ja analüüsitakse anonüümselt ning vastajaid ei ole võimalik uuringu aruandluse põhjal tuvastada. Uuringus osalemine on vabatahtlik ja küsimustikule vastamisega annate teadliku nõusoleku uuringus osalemiseks. Uuringu on heaks kiitnud Tartu Ülikooli inimuuringute eetika komitee (dokumendi nr 330/T-18).

Oleme Teie vastuste eest väga tänulikud, kuna tegemist on esimese praktilise uuringuga piiriülese digiretsepti kohta. Uuring on avatud **3. maini 2021**. Kui Teil on lisaküsimusi, võtke palun ühendust:

Reelika Jõgi

Proviisoriõppe tudeng

Farmaatsia instituut, Tartu Ülikool

555 94 769

[reelika.jogi@ut.ee](mailto:reelika.jogi@ut.ee)

Juhul, kui Teil on küsimusi enda kui uuritava õiguste kohta, siis palume pöörduda Tartu Ülikooli inimuuringute eetika komitee poole, e-post eetikakomitee@ut.ee, tel 737 6215 ning kui Teil on küsimusi andmekaitse osas, siis palume pöörduda Andmekaitse Inspektsiooni poole e-post: info@aki.ee, tel 5620 2341.

1. Kui sageli Te väljastasite piiriüleseid digiretsepte **keskmiselt 2020. aastal**?

Palun valige **ainult üks** järgnevatest:

- Igapäevaselt või peaaegu igapäevaselt
- Ligikaudu korra nädalas
- Ligikaudu paar korda kuus
- Ligikaudu korra kuus
- Vähem kui korra kuus
- Ma ei väljastanud piiriüleseid digiretsepte 2020. aastal

2. Kas Te tunnete, et olete saanud piiriüleste digiretseptide väljastamiseks piisavalt väljaõpet?

Palun valige **ainult üks** järgnevatest:

- Jah
- Ei
- Ma ei ole saanud väljaõpet

3. Millist väljaõpet Te saite? Võite valida mitu vastusevarianti.

Palun valige **kõik**, mis sobib:

- Auditoorne seminar/koolitus
- Veebiseminar
- Koolitusvideo
- Otsisin iseseisvalt teavet veebilehtedelt
- Juhised e-mailil/paberil
- Muu: __________

4. Millistel piiriülese digiretsepti teemadel vajaksite täiendavat väljaõpet?

Kirjutage vastus siia: _________________________________________

5. Kas Te oleksite vajanud piiriülese digiretsepti väljastamiseks väljaõpet?

Palun valige **ainult üks** järgnevatest:

- Jah
- Ei

6. Millistel piiriülese digiretsepti teemadel oleksite vajanud väljaõpet?

Kirjutage vastus siia: _________________________________________

7. Kas Teil on vajadusel juurdepääs piiriülese digiretsepti väljastamise juhistele?

Palun valige **ainult üks** järgnevatest:

- Jah
- Ei
- Ma ei tea

8. Millistele juhistele on Teil ligipääs?

Kirjutage vastus siia: _________________________________________

9. Kas Teie arvates on piiriülese digiretsepti süsteem isikuandmete kaitse seisukohalt turvaline?

Palun valige **ainult üks** järgnevatest:

- Jah
- Ei

10. Millised teemad on problemaatilised?

Kirjutage vastus siia: _________________________________________

11. Kas Te teavitate patsienti piiriülest digiretsepti väljastades isikuandmete töötlemisest Eestis?

Palun valige **ainult üks** järgnevatest:

Alati

Sageli

Harva

Mitte kunagi

12. Mis põhjus(t)el Te ei teavita patsienti isikuandmete töötlemisest Eestis?

Kirjutage vastus siia: _________________________________________

13. Kas Teil on tekkinud probleeme patsiendi isikutuvastamisega piiriülese digiretsepti väljastamisel?

Palun valige **ainult üks** järgnevatest:

Alati

Sageli

Harva

Mitte kunagi

14. Milliseid probleeme on Teil tekkinud patsiendi isikutuvastamisel?

Kirjutage vastus siia: _________________________________________

15. Kui sageli on esinenud piiriülestes digiretseptides **ebaselgusi või vigu** **(näiteks seoses manustamisjuhiste, ravimi tugevuse või ATC-koodiga),** mis on vajanud väljastamisel täpsustamist?

Palun valige **ainult üks** järgnevatest:

- Alati
- Sageli
- Harva
- Mitte kunagi

16. Millised ebaselgusi või vigu on piiriülestes digiretseptides esinenud?

Palun valige **kõik**, mis sobib:

- Erinevused ATC-koodides Eesti/Soome või muu riigi vahel
- Vale ravim
- Ravimi vale tugevus
- Vale ravimvorm
- Ravimi vale kogus
- Ebaselged või valed manustamisjuhised
- Manustamisjuhised puuduvad
- Lapse (alla 12a) kaal puudub
- Muu: __________

17. Milline on Teie arvamus järgnevate väidete kohta? Valige igale väitele sobiv vastusevariant skaalalt.

|  | Nõustun täielikult | Pigem nõustun | Pigem ei nõustu | Ei nõustu üldse | Ei oska vastata |
| --- | --- | --- | --- | --- | --- |
|  |  |  |  |  |  |
| Enne apteeki tulekut on patsientidel piisavalt teavet piiriülese digiretsepti kohta | ○ | ○ | ○ | ○ | ○ |
| Ravimite nomenklatuur on piiriülese digiretsepti väljastamiseks piisav | ○ | ○ | ○ | ○ | ○ |
| Piiriülene digiretsept tagab ravimite ohutu kasutamise | ○ | ○ | ○ | ○ | ○ |
| Piiriülese digiretseptiga patsiendi raviminõustamine on vajalik | ○ | ○ | ○ | ○ | ○ |
| Piiriülese digiretseptiga patsienti on keelebarjääri tõttu keeruline nõustada | ○ | ○ | ○ | ○ | ○ |
| Ainult patsiendi emakeeles olevad annustamisjuhised muudavad piiriülese digiretsepti korral raviminõustamise keeruliseks | ○ | ○ | ○ | ○ | ○ |
| Ravimite koostoimeid on piiriülese digiretseptiga ravimit väljastades lihtne jälgida | ○ | ○ | ○ | ○ | ○ |
| Piiriülene digiretsept on parandanud patsientide ravimite kättesaadavust | ○ | ○ | ○ | ○ | ○ |

18. Kui Teil on ülaltoodud väidetega seonduvaid kommentaare, siis võite need jätta siia:

Kirjutage vastus siia: _________________________________________

19. Kui sageli on piiriülese digiretsepti väljastamisel esinenud probleeme ravimite kättesaadavusega?

Palun valige **ainult üks** järgnevatest:

Alati

Sageli

Harva

Mitte kunagi

20. Millised ravimite kättesaadavuse probleemid on piiriülese digiretsepti väljastamisel esinenud?

Palun valige **kõik**, mis sobib:

- Välja kirjutatud ravim on hetkel apteegist otsas
- Välja kirjutatud sama toimeainega ravim ei ole Eesti turul saadaval
- Vastav ravimi tugevus ei ole Eesti turul saadaval
- Vastav ravimvorm ei ole Eesti turul saadaval
- Vastav ravimipakendi suurus ei ole Eesti turul saadaval
- Muu: __________

21. Millist apteegitarkvara Te oma töös kasutate?

Palun valige **ainult üks** järgnevatest:

- NOOM
- Hansasoft
- Muu: __________

22. Mis on Teie arvamus järgmiste väidete kohta? Palun vastake **eelmises küsimuses valitud apteegitarkvara** põhjal ning valige igale väitele kõige sobivam vastusevariant skaalalt.

|  | Nõustan täielikult | Pigem nõustun | Pigem ei nõustu | Ei nõustu  üldse | Ei oska vastata |
| --- | --- | --- | --- | --- | --- |
|  |  |  |  |  |  |
| Piiriülese digiretsepti rakendust on lihtne kasutada | ○ | ○ | ○ | ○ | ○ |
| Piiriülese digiretsepti rakendust on lihtne kasutama õppida | ○ | ○ | ○ | ○ | ○ |
| Piiriülese digiretsepti rakendus on paindlik | ○ | ○ | ○ | ○ | ○ |
| Piiriülese digiretsepti rakendus on arusaadav | ○ | ○ | ○ | ○ | ○ |
|  |  |  |  |  |  |

23. Kui sageli olete piiriülese digiretsepti rakenduse kasutamisel kogenud **tehnilisi probleeme**, mis on takistanud või aeglustanud retsepti väljastamist?

Palun valige **ainult üks** järgnevatest:

- Alati
- Sageli
- Harva
- Mitte kunagi

24. Kas Te olete kunagi tehnilise probleemi tõttu olnud sunnitud jätma piiriülese digiretsepti väljastamata?

Palun valige **ainult üks** järgnevatest:

Jah

Ei

25. Palun täpsustage, milliseid tehnilisi probleeme Te olete kogenud piiriülese digiretsepti väljastamisel?

Kirjutage vastus siia: _________________________________________

26. Kas need tehnilised probleemid on praeguseks lahendatud?

Kirjutage vastus siia: _________________________________________

27. Kust Te saate vajadusel tehnilist abi?

Kirjutage vastus siia: _________________________________________

28. Kes on Teie arvates **tüüpiline piiriülese digiretseptiga patsient**?

Palun valige **ainult üks** järgnevatest:

- Soomlane, kes on Eestit külastamas
- Soomlane, kes töötab või elab Eestis
- Eestlane, kellel on Soome ID-kaart
- Muu
- Ei oska öelda

29. Mis on Teie arvates piiriülese digiretsepti peamised tugevused?

Kirjutage vastus siia: _________________________________________

30. Mis on Teie arvates peamised probleemid või arendamist vajavad valdkonnad piiriülese digiretsepti juures?

Kirjutage vastus siia: _________________________________________

31. Kui rahul olete piiriülese digiretseptiga tervikuna?

Ei ole üldse rahul Väga rahul

1 2 3 4 5

32. Mis piirkonnas asub apteek, kus Te töötate?

Palun valige **ainult üks** järgnevatest:

- Tallinn
- Harjumaa (väljaspool Tallinna)
- Tartu ja Tartumaa, Põlvamaa, Võrumaa, Valgamaa
- Lääne-Eesti (Pärnumaa, Läänemaa, Saaremaa, Hiiumaa)
- Virumaa (Lääne-Virumaa, Ida-Virumaa)
- Viljandimaa, Jõgevamaa, Raplamaa ja Järvamaa

33. Kellena Te apteegis töötate?

Palun valige **ainult üks** järgnevatest:

- Farmatseut
- Proviisor
- Proviisor-omanik
- Proviisor-juhataja

34. Kui kaua Te olete jaeapteegis töötanud?

Palun valige **ainult üks** järgnevatest:

- Vähem kui 1 aasta
- 1-5 aastat
- 6-10 aastat
- 11-20 aastat
- Rohkem kui 20 aastat

35. Teie vanus?

Palun valige **ainult üks** järgnevatest:

- ≤ 29
- 30–39
- 40–49
- 50−59
- ≥ 60

36. Milliseid keeli Te valdate suhtlustasemel lisaks eesti keelele?

Palun valige **kõik**, mis sobib:

- Inglise
- Vene
- Saksa
- Soome
- Muu(d)

37. Muud mõtted ja kommentaarid piiriülese digiretsepti või antud küsimustiku kohta:

Kirjutage vastus siia: _________________________________________

Aitäh, et leidsite aega küsimustikule vastamiseks! Kui Teil tekib täiendavaid küsimusi või kommentaare, võtke palun ühendust:

Reelika Jõgi

Proviisoriõppe tudeng

Farmaatsia instituut, Tartu Ülikool

555 94 769

[reelika.jogi@ut.ee](mailto:reelika.jogi@ut.ee)
