# Supplementary material for: Implementation of European Cross-border Electronic Prescription and Electronic Dispensing Service: Cross-sectional Survey
Source: J Med Internet Res. 2023 Apr 4;25:e42453. doi: 10.2196/42453 (PMC10132001; doi:10.2196/42453)
Supplement: Multimedia Appendix 2 [file jmir_v25i1e42453_app2.docx]

**Appendix 2 - Kyselytutkimus farmaseuttiselle henkilöstölle rajat ylittävästä sähköisestä reseptistä**

Arvoisat farmaseutit, proviisorit ja apteekkarit,

Tervetuloa mukaan tutkimukseen ”Rajat ylittävä sähköinen lääkkeen määrääminen ja toimittaminen – ensimmäiset tulokset Virosta ja Suomesta” (Cross-border e-prescribing and e-dispensing – the first results from Estonia and Finland). Tämä kysely on tarkoitettu vain niille **farmaseuteille, proviisoreille ja apteekkareille, joilla on kokemusta sähköisten eurooppalaisten reseptien toimittamisesta (myöhemmin tässä kyselyssä käytetään ilmaisua rajat ylittävä resepti)**.

Direktiivin 2011/24/EU potilaiden oikeuksien soveltamisesta rajat ylittävässä terveydenhuollossa mukaan EU-kansalaiset voivat päästä turvalliseen ja laadukkaaseen terveydenhoitoon missä tahansa EU-maassa ja saada korvauksen ulkomailla saamastaan hoidosta omassa kotimaassaan. Vuonna 2014 kehitettiin ohjeet sähköisten reseptien vaihtoon maiden välillä. Reseptitiedon vaihto on osa hanketta, jonka 23 EU-maata aloitti vuonna 2017. Hankkeen tavoitteena oli varmistaa sähköisen tiedon avulla entistä laadukkaampi terveydenhuolto ja lääkkeiden saatavuus.

**Tämän kyselytutkimuksen tavoitteena on tutkia farmaseuttisen henkilöstön kokemuksia rajat ylittävien reseptien toimittamisesta ja ymmärtää rajat ylittävän reseptin vaikutuksia lääkkeiden saatavuuteen ja turvalliseen käyttöön.** Tutkimuksen tuloksia voidaan käyttää kehittämään rajat ylittävän reseptin järjestelmiä. Tässä hankkeessa on mukana sekä suomalaisia että virolaisia farmaseutteja ja proviisoreja. Kyselytutkimuksen rahoittaa Kansaneläkelaitos (Kela) ja tutkimus toteutetaan yhteistyössä Itä-Suomen yliopiston, Tarton yliopiston, Kelan ja Viron lääkeviranomaisen kanssa.

Kyselyn täyttäminen kestää noin 15 minuuttia. Vastaa kysymyksiin valitsemalla sinusta sopivin vaihtoehto tai kirjoita vastaus sille varattuun tilaan.

Kyselyn vastaukset kerätään ja analysoidaan anonyymisti, eikä yksittäistä vastaajaa pysty tunnistamaan tutkimuksen raportoinnista. Tutkimukseen osallistuminen on vapaaehtoista ja kyselyyn vastaamista pidetään tietoisena suostumuksena osallistumiseen. Tutkimukseen liittyvästä henkilötietojen käsittelystä löydät tietoa [täältä](https://studentuef-my.sharepoint.com/personal/sjtimone_uef_fi/_layouts/15/onedrive.aspx?). Tarton yliopiston tutkimuseettinen toimikunta on antanut tutkimuksesta myönteisen lausunnon (lausunto 330/T-18).

Olemme kiitollisia vastauksestasi, sillä tämä on ensimmäinen tutkimus rajat ylittävästä sähköisestä reseptistä. Kysely on avoinna **03.5.2021** saakka. Jos sinulla on kysymyksiä, ota yhteyttä:

Johanna Timonen

Yliopistotutkija, dosentti, FaT

Farmasian laitos, Itä-Suomen yliopisto

040 355 3881

johanna.timonen@uef.fi

1. Kuinka usein **keskimäärin** henkilökohtaisesti toimitit rajat ylittäviä reseptejä **vuonna 2020?**

Valitse **vain yksi** seuraavista:

- Päivittäin tai lähes päivittäin
- Noin kerran viikossa
- Muutaman kerran kuukaudessa
- Noin kerran kuukaudessa
- Harvemmin kuin kuukausittain
- En toimittanut rajat ylittäviä reseptejä lainkaan vuonna 2020

2. Oletko mielestäsi saanut riittävästi koulutusta rajat ylittävän reseptin toimittamiseen?

Valitse **vain yksi** seuraavista:

- Kyllä
- Ei
- En saanut mitään koulutusta

3. Millaista koulutusta sait?

Valitse **kaikki,** jotka soveltuvat:

- Rajat ylittävän reseptin toimittaminen apteekissa – etäyhteysseminaari, THL
- Power point -diat THL/Kanta-palvelut
- Koulutusvideot
- Itsenäinen tiedon hankinta verkkosivuilta
- Sähköpostilla/paperilla annetut ohjeet
- Muu: __________

4. Mistä aiheista rajat ylittävässä reseptissä tarvitsisit enemmän koulutusta?

Vastauksesi: ________________________________________________

5. Olisitko tarvinnut jotain koulutusta rajat ylittävän reseptin toimittamiseen?

Valitse **vain yksi** seuraavista:

- Kyllä
- Ei

6. Mistä aiheista rajat ylittävässä reseptissä olisit tarvinnut koulutusta?

Vastauksesi: ________________________________________________

7. Pääsetkö tarvittaessa katsomaan ohjeita rajat ylittävän reseptin toimittamisesta?

Valitse **vain yksi** seuraavista:

- Kyllä
- Ei
- En tiedä

8. Mitä ohjeita pääset katsomaan?

Vastauksesi: ________________________________________________

9. Onko Rajat ylittävä resepti -palvelu mielestäsi tietosuojan näkökulmasta turvallinen?

Valitse **vain yksi** seuraavista:

- Kyllä
- Ei

10. Mitkä asiat ovat ongelmallisia?

Vastauksesi: ________________________________________________

11. Kun toimitat rajat ylittävää reseptiä, kerrotko asiakkaalle henkilötietojen käsittelystä Suomessa?

Valitse **vain yksi** seuraavista:

Aina

Usein

Harvoin

En koskaan

12. Missä tilanteissa et kerro asiakkaalle henkilötietojen käsittelystä Suomessa?

Vastauksesi: ________________________________________________

13. Kun toimitat rajat ylittävää reseptiä, onko asiakkaan henkilöllisyyden tunnistamisessa ollut ongelmia?

Valitse **vain yksi** seuraavista:

Aina

Usein

Harvoin

Ei koskaan

14. Millaisia ongelmia rajat ylittävällä reseptillä asioivan asiakkaan henkilöllisyyden tunnistamisessa on ollut?

Vastauksesi: ________________________________________________

15. Kuinka usein toimittamissanne rajat ylittävissä resepteissä on ollut **epäselvyyksiä tai virheitä (esim. liittyen lääkkeen annostusohjeeseen, vahvuuteen tai ATC-koodiin),** jotka vaativat asian selvittelyä kesken reseptintoimituksen?

Valitse **vain yksi** seuraavista:

- Aina
- Usein
- Harvoin
- Ei koskaan

16. Millaisia epäselvyyksiä tai virheitä rajat ylittävissä resepteissä on ollut?

Valitse **kaikki,** jotka soveltuvat:

- Eroavuudet Suomen tai toisen maan ATC-koodeissa
- Väärä lääke
- Väärä vahvuus
- Väärä lääkemuoto
- Lääkkeen kokonaismäärä virheellinen
- Epäselvä tai väärä annostusohje
- Annostusohje puuttuu kokonaan
- Lapsen (alle 12 v) paino puuttuu
- SIC! merkintä puuttuu poikkeavassa annostusohjeessa tai käyttötarkoituksessa
- Muu: __________

17. Mikä on mielipiteesi seuraavista väittämistä? Valitse sopivin vastausvaihtoehto jokaiseen väittämään.

|  | Täysin samaa mieltä | Jokseenkin samaa mieltä | Jokseenkin eri mieltä | Täysin eri mieltä | En osaa sanoa |
| --- | --- | --- | --- | --- | --- |
|  |  |  |  |  |  |
| Asiakkaat saavat riittävästi tietoa rajat ylittävästä reseptistä muista lähteistä ennen apteekkiin saapumista. | ○ | ○ | ○ | ○ | ○ |
| Lääkevalikoima soveltuu rajat ylittäville resepteille. | ○ | ○ | ○ | ○ | ○ |
| Rajat ylittävä resepti varmistaa turvallisen lääkkeiden käytön. | ○ | ○ | ○ | ○ | ○ |
| Rajat ylittävillä resepteillä asioivien asiakkaiden lääkeneuvonta on tarpeellista. | ○ | ○ | ○ | ○ | ○ |
| On vaikeaa neuvoa rajat ylittävällä reseptillä asioivaa asiakasta kielimuurin takia. | ○ | ○ | ○ | ○ | ○ |
| Annosohjeet pelkästään asiakkaan omalla kielellä vaikeuttavat rajat ylittävällä reseptillä asioivien asiakkaiden lääkeneuvontaa. | ○ | ○ | ○ | ○ | ○ |
| On helppo havaita lääkkeiden yhteisvaikutuksia rajat ylittävistä resepteistä. | ○ | ○ | ○ | ○ | ○ |
| Rajat ylittävä resepti on parantanut potilailla lääkitysten saavutettavuutta. | ○ | ○ | ○ | ○ | ○ |

18. Jos sinulla on kommentteja edellä mainittuihin väittämiin liittyen, voit jättää ne tähän

Vastauksesi: ________________________________________________

19. Miten usein rajat ylittävissä resepteissä on ollut vaikeuksia lääkkeiden saatavuudessa?

Valitse **vain yksi** seuraavista:

- Aina
- Usein
- Harvoin
- Ei koskaan

20. Millaisia vaikeuksia rajat ylittävissä resepteissä on ollut lääkkeiden saatavuudessa? Voit valita useita vastausvaihtoehtoja.

Valitse **kaikki,** jotka soveltuvat:

- Vastaava lääke on loppu apteekin varastosta
- Vastaavaa lääkettä, jossa on sama vaikuttava aine, ei ole saatavilla Suomen markkinoilta
- Vastaavaa lääkettä, jossa on sama vahvuus, ei ole saatavilla Suomen markkinoilta
- Vastaavaa lääkettä, jossa on sama lääkemuoto, ei ole saatavilla Suomen markkinoilta
- Lääkkeen pakkauksen vastaavaa kokoa ei ole saatavilla Suomen markkinoilta
- Muu: __________

21. Mikä apteekkijärjestelmä on käytössä apteekissa, jossa työskentelet?

Valitse **vain yksi** seuraavista:

- Maxx
- Salix
- PD3

22. Mitä mieltä olet seuraavista väittämistä? Vastaa kysymykseen **perustuen apteekkijärjestelmään, jonka valitsit edellisessä kysymyksessä**. Valitse sopivin vastausvaihtoehto jokaiseen väittämään.

|  | Täysin samaa mieltä | Jokseenkin samaa mieltä | Jokseenkin eri mieltä | Täysin eri mieltä | En osaa sanoa |
| --- | --- | --- | --- | --- | --- |
|  |  |  |  |  |  |
| Rajat ylittävä resepti -palvelu on helppo käyttää | ○ | ○ | ○ | ○ | ○ |
| Rajat ylittävä resepti -palvelun käyttö oli helppo oppia | ○ | ○ | ○ | ○ | ○ |
| Rajat ylittävä resepti -palvelu on joustava käyttää | ○ | ○ | ○ | ○ | ○ |
| Rajat ylittävä resepti -palvelun käyttö on ymmärrettävää | ○ | ○ | ○ | ○ | ○ |
|  |  |  |  |  |  |

23. Kuinka usein sinulla on ollut Rajat ylittävän resepti -palvelun käytössä **tekninen häiriö**, joka on vaikeuttanut/hidastanut reseptin toimittamista?

Valitse **vain yksi** seuraavista:

- Aina
- Usein
- Harvoin
- Ei koskaan

24. Onko tekninen häiriö koskaan estänyt rajat ylittävän reseptin toimittamista?

Valitse **vain yksi** seuraavista:

Kyllä

Ei

25. Millaisia teknisiä ongelmia olet kokenut?

Vastauksesi: ________________________________________________

26. Ovatko kokemasi tekniset ongelmat nyt ratkenneet?

Vastauksesi: ________________________________________________

27. Mistä voit tarvittaessa saada teknistä apua?

Vastauksesi: ________________________________________________

28. Kuka mielestäsi **yleisimmin** on asiakas, joka asioi rajat ylittävällä reseptillä?

Valitse **vain yksi** seuraavista:

- Virolainen matkailija
- Virolainen, joka työskentelee tai asuu Suomessa
- Muu
- En osaa sanoa

29. Mitkä ovat mielestäsi rajat ylittävän reseptin keskeisimmät edut?

Vastauksesi: ________________________________________________

30. Mitkä ovat mielestäsi rajat ylittävän reseptin keskeisimmät ongelmat/kehittämiskohteet?

Vastauksesi: ________________________________________________

31. Kuinka tyytyväinen olet rajat ylittävään reseptiin kokonaisuudessaan? Valitse sopivaksi katsomasi vastausvaihtoehto.

En lainkaan Hyvin

tyytyväinen tyytyväinen

1 2 3 4 5

32. Apteekin, jossa työskentelet, sijainti?

Valitse **vain yksi** seuraavista:

- Helsingin kaupunki
- Muu pääkaupunkiseutu (Espoo, Vantaa, Kauniainen)
- Muu alue Etelä-Suomi
- Länsi- ja Sisä-Suomi
- Lounais-Suomi
- Itä-Suomi
- Pohjois-Suomi
- Lappi

33. Mikä on toimesi apteekissa?

Valitse **vain yksi** seuraavista:

- Farmaseutti
- Proviisori
- Apteekin hoitaja
- Apteekkari

34. Kuinka pitkään olet työskennellyt apteekissa yhteensä?

Valitse **vain yksi** seuraavista:

- Alle vuoden
- 1–5 vuotta
- 6–10 vuotta
- 11–20 vuotta
- Yli 20 vuotta

35. Sukupuolesi?

Valitse **vain yksi** seuraavista:

- Nainen
- Mies
- Muu
- En halua kertoa

36. Ikäsi?

Valitse **vain yksi** seuraavista:

- ≤ 29
- 30–39
- 40–49
- 50–59
- ≥ 60

37. Millä kielillä pystyt kommunikoimaan Suomen lisäksi?

Valitse **kaikki,** jotka soveltuvat:

- Englanti
- Ruotsi
- Venäjä
- Ranska
- Saksa
- Viro
- Muu

38. Muita ajatuksia tai kommentteja rajat ylittävästä reseptistä tai tästä kyselystä

Vastauksesi: ________________________________________________

Kiitos osallistumisesta tähän tutkimukseen! Jos sinulla on kysymyksiä tai muuta huomautettavaa, voit olla yhteydessä:

Johanna Timonen

Yliopistotutkija, dosentti, FaT

Farmasian laitos, Itä-Suomen yliopisto

040 355 3881

[johanna.timonen@uef.fi](mailto:johanna.timonen@uef.fi)
