# Supplementary material for: Implementation of European Cross-border Electronic Prescription and Electronic Dispensing Service: Cross-sectional Survey
Source: J Med Internet Res. 2023 Apr 4;25:e42453. doi: 10.2196/42453 (PMC10132001; doi:10.2196/42453)
Supplement: Multimedia Appendix 3 [file jmir_v25i1e42453_app3.docx]

**Appendix 3 - Questionnaire study for pharmacists regarding cross-border e-prescriptions (CBeP)**

Welcome to the study “A cross-border e-prescribing and e-dispensing – the first results from Estonia and Finland”. This questionnaire is targeted **only for those pharmacists who have experience dispensing cross-border e-prescriptions.**

With Directive 2011/24/EU on patients’ rights in cross-border healthcare EU citizens can access safe and high-quality healthcare in any EU country and to be reimbursed for care abroad by their home country. In 2014 guidelines on e-prescriptions dataset for electronic exchange were developed. Exchange of prescription data is a part of a project of 23 EU countries started in 2017 with aim to ensure healthcare with increased quality and availability of medicinal products via electronic data.

**The aim of this questionnaire study is to explore pharmacists' experiences in dispensing cross-border e-prescriptions, and to understand the impact of a cross-border e-prescription on the availability and safe use of medicines.** The results of the study can be used to develop a cross-border e-prescriptions system. In this project, both Estonian and Finnish pharmacists are involved. This questionnaire study is funded by Kansaneläkelaitos (Kela) and the study is carried out in cooperation of University of Eastern Finland, University of Tartu, Kela and Estonian State Agency of Medicines.

It takes approximately 15 minutes to fill in the questionnaire. Answer the questions by choosing the most suitable answer(s) or write the answer in the space provided.

The answers of this questionnaire are collected and analysed anonymously, and the individual respondents cannot be identified by the reporting of the study or in the final study report. Participating in this study is voluntary and answering to the questionnaire is regarded as informed consent to participate. Link to privacy statement can be found here (FIN). The study has been approved by the Research Ethics Committee of the University of Tartu (approval 330/T-18).

We are very grateful for your answer, as this is the first study on cross-border e-prescription in practice. The survey is open until **3rd May 2021.** If you have any further questions, please contact:

FIN:

Johanna Timonen

Senior researcher (Yliopistotutkija)

School of pharmacy, University of Eastern Finland (Farmasian laitos, Itä-Suomen yliopisto)

040 355 3881

[johanna.timonen@uef.fi](mailto:johanna.timonen@uef.fi)

EST:

Reelika Jõgi

Pharmacy student

Institute of Pharmacy, University of Tartu

555 94 769

[reelika.jogi@ut.ee](mailto:reelika.jogi@ut.ee)

1. How often did you personally dispense CBePs **on average in 2020**?

Please select **only one** of the following:

- Daily or almost daily
- About once a week
- A few times a month
- About once a month
- Less than once a month
- I did not dispense CBeP in 2020

2. Do you feel you have received sufficient training for CBeP dispensing?

Please select **only one** of the following:

- Yes
- No
- I did not receive any training

3. What kind of training did you receive? You may choose several options.

FIN:

- Face-to-face seminar
- PowerPoint slides from THL and Kanta services
- Training videos
- Independent retrieval of information
- Instructions via email or paper
- Other, please specify: __________

EST:

- Face-to-face seminar
- Web-based seminar
- Training videos
- Independent retrieval of information
- Instructions on email or paper
- Other, please specify: __________

4. On what topics in CBePs would you need more training on?

Answer: _________________________________________

5. Would you have needed some training for CBeP dispensing?

Please select **only one** of the following:

- Yes.
- No

6. On what topics in CBePs would you have needed training on?

Answer: _________________________________________

7. Do you have access to guidelines for CBeP dispensing if needed?

Please select **only one** of the following:

- Yes
- No
- I don’t know

8. What guidelines do you have access to?

Answer: _________________________________________

9. Do you think the CBeP system is safe from the standpoint of personal data protection?

Please select **only one** of the following:

- Yes
- No

10. What topics are problematic?

Answer: _________________________________________

11. When dispensing the CBeP, do you inform the customer about the processing of personal data in Finland/Estonia?

Please select **only one** of the following:

Always

Often

Rarely

Never

12. In what situations do you not inform the customer about the processing of personal data in Finland/Estonia?

Answer: _________________________________________

13. When dispensing the CBeP, have there been problems identifying the customer?

Please select **only one** of the following:

Always

Often

Rarely

Never

14. What kind of problems have occurred with identifying the customer with CBeP?

Answer: _________________________________________

15. How often have there been **ambiguities or errors in CBePs** (e.g., in relation to dosage instructions, drug strength or ATC code), that have required clarification during dispensing process?

Please select **only one** of the following:

- Always
- Often
- Rarely
- Never

16. What kinds of ambiguities or errors have there been in the CBePs?

Please select **all** that apply:

- Differences between ATC codes between countries
- Incorrect medication
- Incorrect strength
- Incorrect pharmaceutical form
- Incorrect total amount of medication
- Unclear or incorrect dosage instructions
- Missing dosage instructions
- Weight of child (aged <12) missing
- Missing notation of exceptional dosage instructions or exceptional purpose of use (FIN)
- Other, please specify: __________

17. What is your opinion on the following statements? Choose the most suitable alternative for each statement.

|  | I  fully  agree | I  agree somewhat | I  disagree somewhat | I  fully disagree | I  don’t  know |
| --- | --- | --- | --- | --- | --- |
|  |  |  |  |  |  |
| Customers receive sufficient information about CBeP from other sources of information before coming to the pharmacy | ○ | ○ | ○ | ○ | ○ |
| The drug nomenclature is sufficient for CBePs | ○ | ○ | ○ | ○ | ○ |
| CBeP ensures the safe use of medications | ○ | ○ | ○ | ○ | ○ |
| Medicine counselling the customers with CBePs is necessary | ○ | ○ | ○ | ○ | ○ |
| It is difficult to counsel the customer with CBeP due to language barrier | ○ | ○ | ○ | ○ | ○ |
| Dosage instructions only in the customer’s own language does complicate medicine counselling the patients with CBeP | ○ | ○ | ○ | ○ | ○ |
| It is easy to monitor drug interactions with CBeP | ○ | ○ | ○ | ○ | ○ |
| CBeP has improved patients’ access to medications | ○ | ○ | ○ | ○ | ○ |

18. If you have any comments related to the statements above, you can leave them here:

Answer: _________________________________________

19. How often there have been problems with CBePs in the availability of medication?

Please select **only one** of the following:

Always

Often

Rarely

Never

20. What kind of problems have there been with CBePs in the availability of medication? You may choose several options.

Please select **all** that apply:

- Pharmacy currently does not have the corresponding medication in stock
- Corresponding medication with the same active ingredient is not available in the market
- Corresponding medication with the same strength is not available in the market
- Corresponding medication with the same formulation is not available in the market
- Equivalent package size is not available in the market
- Other, please specify: __________

21. What pharmacy system is used at the pharmacy where you work?

Please select **only one** of the following:

1. NOOM / Maxx
2. Hansasoft / Salix
3. Other / PD3

22. What is your opinion on the following statements **based on the application selected in the previous question**? Choose the most suitable alternative for each statement.

|  | I  fully  agree | I  somewhat  agree | I  somewhat  disagree | I  fully  disagree | I  don’t  know |
| --- | --- | --- | --- | --- | --- |
|  |  |  |  |  |  |
| The CBeP application is easy to use | ○ | ○ | ○ | ○ | ○ |
| The CBeP application was easy to learn to use | ○ | ○ | ○ | ○ | ○ |
| The CBeP application is flexible | ○ | ○ | ○ | ○ | ○ |
| The CBeP application is understandable | ○ | ○ | ○ | ○ | ○ |
|  |  |  |  |  |  |

23. How often have you experienced **a technical problem in using CBeP system** that has hindered/slowed dispensing of a prescription?

Please select **only one** of the following:

- Always
- Often
- Rarely
- Never

24. Have you ever been unable to dispense a CBeP due to a technical problem?

Please select **only one** of the following:

Yes

No

25. Can you please specify, what kind of technical problems have you experienced?

Answer: _________________________________________

26. Are these technical problems solved by now?

Answer: _________________________________________

27. From where can you receive technical help if needed?

Answer: _________________________________________

28. In your opinion, who are **most of the customers** with CBePs?

Please select **only one** of the following:

FIN:

- Estonian tourists
- Estonians working or living in Finland
- Other
- I don’t know

EST:

- Finnish tourists
- Finns working or living in Estonia
- Estonian with Finnish ID-card
- Other
- I don’t know

29. In your opinion, what are the main benefits of CBeP?

Answer: _________________________________________

30. In your opinion, what are the main problems/areas needing development in CBeP?

Answer: _________________________________________

31. How satisfied are you with CBeP as a whole? Choose the most suitable alternative.

Not satisfied Very

at all satisfied

1 2 3 4 5

32. The pharmacy where you work is located in? (click to open menu)

Please select **only one** of the following:

FIN:

- Helsingin kaupunki (city of Helsinki)
- Surrounding metropolitan area (Espoo, Vantaa, Kauniainen)
- Rest of the region South Finland)
- Western or central Finland
- South-western Finland
- Eastern Finland
- Northern Finland
- Lapland

EST:

- Tallinn
- Harjumaa (outside of Tallinn)
- Tartu ja Tartumaa, Põlvamaa, Võrumaa, Valgamaa – south-eastern Estonia
- Lääne-Eesti (Pärnumaa, Läänemaa, Saaremaa, Hiiumaa) - western Estonia
- Virumaa (Lääne-Virumaa, Ida-Virumaa) - eastern and northern Estonia
- Viljandimaa, Jõgevamaa, Raplamaa ja Järvamaa - central Estonia

33. What is your position within the pharmacy?

Please select **only one** of the following:

- Dispenser or assistant pharmacist
- Pharmacist
- Pharmacy manager
- Pharmacy owner

34. For how long have you worked in a community pharmacy in total?

Please select **only one** of the following:

- Less than a year
- 1 to 5 years
- 6 to 10 years
- 11 to 20 years
- More than 20 years

35. Your sex? (FIN)

Please select **only one** of the following:

- Female
- Male
- Other
- Would not like to specify

36. Your age?

Please select **only one** of the following:

- ≤ 29
- 30 to 39
- 40 to 49
- 50 to 59
- ≥ 60

37. In what languages are you able to communicate in addition to Estonian/Finnish? You may choose several options.

EST:

- English
- Russian
- German
- Finnish
- Other

FIN:

- English
- Swedish
- Russian
- French
- German
- Estonian
- Other

38. Other thoughts and comments regarding CBeP or this questionnaire:

Answer: _________________________________________

Thank you for participating in the study! If you have any additional questions/remarks, please contact:

FIN:

Johanna Timonen

Senior researcher (Yliopistotutkija)

School of pharmacy, University of Eastern Finland (Farmasian laitos, Itä-Suomen yliopisto)

040 355 3881

[johanna.timonen@uef.fi](mailto:johanna.timonen@uef.fi)

EST:

Reelika Jõgi

Pharmacy student

Institute of Pharmacy, University of Tartu

555 94 769

[reelika.jogi@ut.ee](mailto:reelika.jogi@ut.ee)
